# Physical Activity, Sedentary Behavior, Cardiorespiratory Fitness and Metabolic Syndrome in Adolescents: Systematic Review and Meta-Analysis of Observational Evidence

**DOI:** 10.1371/journal.pone.0168503

**Published:** 2016-12-20

**Authors:** Raphael Gonçalves de Oliveira, Dartagnan Pinto Guedes

**Affiliations:** 1 Centro de Pesquisa em Ciências da Saúde, Universidade Norte do Paraná (UNOPAR), Londrina, Paraná, Brazil; 2 Centro de Ciências da Saúde, Universidade Estadual do Norte do Paraná (UENP), Jacarezinho, Paraná, Brazil; University of Missouri Columbia, UNITED STATES

## Abstract

**Background:**

Metabolic syndrome (MetS) has been diagnosed in adolescents and among the associated factors are low levels of physical activity, sedentary behavior over long periods and low cardiorespiratory fitness. However, specifically in adolescents, studies present conflicting results. The aim of the present study was to conduct a systematic review and meta-analysis of observational studies, in order to map the association between physical activity, sedentary behavior, cardiorespiratory fitness and MetS in adolescents.

**Methods:**

A search was performed in the databases PubMed, SPORTDiscus, LILACS and the Cochrane Library. For the meta-analysis, the odds ratio (OR) was calculated together with the respective confidence intervals (95% CI), in which the measures of effect were analyzed by dichotomous data (exposure variables) with MetS used as events.

**Results:**

Eighteen studies were included in the meta-analysis. Primary analysis demonstrated that low levels of physical activity (OR = 1.35 [1.03 to 1.79]; p = 0.03) and low cardiorespiratory fitness (OR = 4.05 [2.09 to 7.87]; p < 0.01) were significantly associated with the development of MetS, while for sedentary behavior, represented by screen time > 2 hours/day, a significant association was not identified (OR = 1.20 [0.91 to 1.59]; p = 0.20). Subgroup analyses demonstrated that the association between low physical activity and MetS was dependent on the use of the accelerometry technique (OR = 2.93 [1.56 to 5.47]; p < 0.01). Screen time > 2 hours/day was significantly associated with MetS only on weekends (OR = 2.05 [1.13 to 3.73]; p = 0.02). With respect to cardiorespiratory fitness, a significant association with MetS was found independent of the maximal oxygen uptake (VO_2_max) measurement method.

**Conclusions:**

Low levels of physical activity, low indices of cardiorespiratory fitness and sedentary behavior, represented by screen time > 2 hours/day on weekends, were significantly associated with the development of MetS in adolescence.

## Introduction

Metabolic syndrome (MetS) is defined by a set of factors, which, if altered, considerably increase the risk of developing cardiovascular disease and type 2 diabetes mellitus [1,2]. To identify carriers of MetS, at least three of the following must be present: elevated blood pressure, modified plasma lipids (elevated triglycerides and reduced high-density lipoprotein cholesterol), elevated fasting glucose and excess body fat (especially in the abdominal region) [1–4]. Currently, for the adult population, there is consensus on the risk factors that compose MetS and their respective cut-off points [3,4].

However, this is not the case in adolescents, for whom the diagnostic criteria varies considerably between the different proposals available, making difficult any comparison between studies [5,6]. Despite this limitation, due to the importance and need to track the presence of MetS and its associated factors as early as possible, several studies have been conducted involving the adolescent population [7–27].

Among the factors identified as possible determinants of MetS in adolescents, are insufficient practice of moderate-to-vigorous physical activity, sedentary behavior (e.g., sitting, watching TV and playing video games) and low cardiorespiratory fitness [28]. In adults, systematic reviews and meta-analysis of observational evidence have shown a strong association between low levels of physical activity [29], prolonged sedentary behavior [30] and a higher chance of developing MetS. However, in the case of adolescents, to our knowledge no study has sought to summarize the individual results using meta-analysis procedures.

Specifically in adolescents, some studies have found an inverse association between low levels of physical activity and higher risk of developing MetS [18,19,23,24], however, other studies have not identified any association [11,12,14,16,20,25]. Similarly, sedentary behavior over a long period has been mentioned in some studies as associated with higher chances of developing MetS [14,21,25], while other studies found no association [10,12]. A systematic review [31] indicated that there seems to be evidence that moderate-to-vigorous physical activity is associated with lower risks of adolescents developing MetS. Another systematic review study [32] found that high sedentary behavior, represented by screen time > 2 hours/day, is associated with an increased risk of MetS. However, neither systematic review [31,32] summarized the data using meta-analysis procedures.

On the other hand, in relation to cardiorespiratory fitness, individual studies involving adolescents [8,16,23,24,27] point to an inverse association with MetS, despite the real effect of the association remaining unclear. This variable is of major interest in view of its strong association with metabolic health [28]. Cardiorespiratory fitness tends not to present large measurement variations, while physical activity and sedentary behavior may demonstrate greater intra-subject variability, especially in youth [28].

Another issue is the fact that the majority of studies [7,9,10–15,17,18,20,21,23–26] utilize self reported measures to evaluate physical activity and sedentary behavior, through questionnaires and recall records. Self reported measures can compromise the validity of the collected data, a fact which is amplified in young people due to the difficulty teenagers have to accurately remember the intensity, duration and frequency of physical activity [33].

Thus, the objective of this systematic review and meta-analysis was to summarize the results of observational data presented in case-control, cross-sectional or prospective cohort studies that examined the association between physical activity, sedentary behavior, cardiorespiratory fitness and MetS in adolescents from 10–19 years old.

## Materials and Methods

This study is characterized as a systematic review, accompanied by meta-analysis, according to the PRISMA protocol (S1 Table. PRISMA Checklist) [34,35] and registered in PROSPERO (CRD42016043113). The inclusion criteria were: a) observational studies (case-control, cross-sectional and prospective cohort); b) studies which identified MetS, taking into account criteria adapted for adolescents; c) studies that considered physical activity, sedentary behavior and cardiorespiratory fitness as an exposure variable and MetS as the outcome variable; and d) studies that included adolescents aged 10–19 years.

The exclusion criteria were: a) no observational study design; b) studies with duplicate information already included in other studies; c) studies that did not define MetS, or only considered the risk dimension based on a continuous score; c) MetS not associated with the variables of interest (physical activity, sedentary behavior and cardiorespiratory fitness); d) adult population (> 19 years) or children (<10 years); and e) studies including adolescents with a pathological condition, physical or mental disability.

### Databases & search strategy

The search was conducted in the databases PubMed, SPORTDiscus, LILACS and the Cochrane Library without the use of a filter to limit date of publication or language. The final search took place on May 07, 2016. As the search strategy, the following keywords were selected: (“metabolic syndrome” OR “metabolic syndrome x” OR “syndrome x”) AND (“physical activity” OR “motor activity” OR “sedentary behavior” OR “sedentary activity” OR “sedentary lifestyle” OR “screen time” OR “physical fitness” OR “cardiorespiratory fitness” OR “exercise” OR “sports”) AND (“adolescent” OR “youth” OR “teen” OR “teenager”). The search strategy was adapted for each database when necessary. A manual search taking into account the reference lists of included studies was used to supplement the database search.

### Selection of studies

One reviewer (RGO) conducted the initial search strategy in the databases, extracting titles and abstracts. Subsequently, the selection of studies, analysis and data extraction were conducted independently by two authors (RGO and DPG), based on blindly reading the titles and abstracts. Potentially eligible articles were read in full. Subsequently, the disagreements were resolved by consensus between the two authors who used the same form for data extraction.

### Data extraction

The following were extracted from each study: a) identification of the authors; b) year of publication; c) country of origin; d) sample size; e) gender and age of the subjects; f) study design (case-control, cross-sectional or prospective cohort); g) MetS identification criteria; h) prevalence of MetS; i) indicator used to measure physical activity, sedentary behavior and cardiorespiratory fitness; j) stratification criteria for physical activity, sedentary behavior and cardiorespiratory fitness; k) adjustment variables; and l) principle results.

### Evaluation of the methodological quality of the studies

To evaluate the potential risk of bias and methodological quality of the studies, each study was critically analyzed blindly by the authors using an adapted version of the tool proposed by Downs and Black [36]. This tool is comparable to the Cochrane Collaboration tool for assessing risk of bias, and has often been used in systematic reviews and meta-analyses of observational studies [37–40]. As recommended, the items of the original check list directed to experimental studies and items that did not apply to this study were excluded. The version of the tool used consisted of 10 items wherein, in each item a score was assigned: 1 point (confirmed attribute) or 0 points (unconfirmed attribute). Thus, the studies included in the systematic review and meta-analysis were classified as high (10–9 points), moderate (8–6 points), low (5–4 points) or very low quality (< 4 points). Possible disagreements on the final score were resolved by consensus among the authors.

### Definitions

Physical activity was defined as any bodily movement produced by skeletal muscles that resulted in energy expenditure above resting levels. Since physical activity is a complex behavior, it is typically divided into categories, which are mutually exclusive, such as light, moderate or vigorous intensity [41]. On the other hand, sedentary behavior was defined as activities involving low energy expenditure, equivalent to 1.0 to 1.5 metabolic equivalent units (METs), such as remaining seated or lying down, reading, watching TV, playing video games, or other forms of entertainment based on a screen [42]. Furthermore, cardiorespiratory fitness was defined as the ability of the respiratory and circulatory systems to supply fuel during prolonged activities and eliminate fatigue, translated by the maximal oxygen uptake (VO_2_max) [41].

MetS was defined as a cluster of risk factors for cardiovascular disease and type 2 diabetes mellitus, which included: a) high blood pressure; b) increased triglycerides; c) reduced high-density lipoprotein cholesterol (HDL-c); d) impaired fasting glucose; and e) abdominal obesity [3,4]. The factors were considered independently of the cut-off points established for each of them, which may vary between the different criteria proposed for adolescents [5,6]. To define MetS, different definitions were developed, principally from the criteria adopted by the National Cholesterol Education Program Adult Treatment Panel III (NCEP-ATPIII) [43], with cut-off points adapted to adolescents (eg.: Ford et al. [44], de Ferranti et al. [45], Cook et al. [46], Jolliffe and Janssen [47]), in which any three of the five factors were required to be present. Another definition, created by the International Diabetes Federation (IDF) [48], considers the mandatory presence of abdominal obesity and any other two factors.

Some studies [8,13,15,17,18,20,26] identify MetS using two or more criteria together, which is denominated harmonized criteria [29]. In the present review, MetS was not considered when its identification occurred through the presence of only two risk factors, or when it was considered through a continuous score (Z-score).

### Synthesis of results

The descriptive results of the qualitative synthesis of the studies are presented in tables. For the meta-analysis, the measures of effects were analyzed by dichotomous data (low level of physical activity vs. moderate/high level of physical activity, low screen time vs. high screen time, low cardiorespiratory fitness vs. moderate/high cardiorespiratory fitness) and MetS taken as an event in each category. To verify that there is a greater chance for the development of MetS among the different classifications within the exposure variables, the Odds Ratio (OR) was calculated with a confidence interval of 95% (CI 95%). The OR is 1 when there is no association, being significant only when the 95% CI does not pass the value of 1. The Cochrane Q test for heterogeneity was performed, assuming a statistical significance of p ≤ 0.10. Heterogeneity was also quantified through the I^2^ statistic, where ≤ 40% does not indicate significant heterogeneity, 30–60% indicates moderate heterogeneity, 50–90% indicates high heterogeneity and ≥ 75% indicates considerable heterogeneity [49]. When statistically significant heterogeneities were not identified, the fixed effects model was used; otherwise, we used the random effects models. Statistical significance equivalent to the values of the effect size of the association was considered as p < 0.05. Forest plots were generated for each test ordered by year of publication of the studies. To evaluate the risk of publication bias, a funnel plot was used when there were ≥ 10 studies in the same meta-analysis. All analyses were performed with the program Review Manager (RevMan) [Computer program], version 5.3, Copenhagen: The Nordic Cochrane Centre, The Cochrane Collaboration.

#### Data handling

For statistical calculation, all ORs were converted into their natural logarithms (log), accompanied by the respective standard error (SE). Thus, when necessary, it was possible to reverse the order of the categories for a given variable [50]. Whenever available the most adjusted OR (adjusted statistically for different covariates) was taken directly from the publications [14,17,18,20,23,25]. However, when OR scores were not available in the studies included in the systematic review [9,12,15,19,22], absolute data were taken for the total quantity of volunteers and MetS events in each meta-analysis comparison category. For missing data, the authors of the selected studies [8,10,11,13,16,21,23–27] were contacted on at least three occasions by one of the reviewers (RGO). There was a 64% response rate [8,10,13,16,23–25]. Laurson et al. [8], Fadzlina et al. [10], Múnera et al. [13], Stabelini Neto et al. [16] and McMurray et al. [24] provided the missing data. When the information was not available, estimates were used to obtain the missing data, converting the percentage values (total quantity of volunteers and MetS events in each meta-analysis comparison category) given in the original publication [21,26,27] to absolute values.

Thus, only one study selected in the systematic review [11] was not included in the meta-analysis of physical activity due to lack of data; and two others [23,25] had sufficient information for the meta-analysis on physical activity, but not for sedentary behavior, so could be included only in the first analysis (physical activity). One publication [22] provided the data on the total number of participants in each category of interest, however, the quantity of MetS events did not allow actual calculation of OR. Furthermore, one case-control study [7], was not comparable to the others in the quantitative synthesis (meta-analysis), given its sample selection feature (intentionally included 32 obese young people with MetS). Thus, three studies [7,11,22] were not included in the meta-analysis and could only be considered in the qualitative synthesis.

#### Primary analysis

In the primary analysis all studies with available data were considered, regardless of methodological quality. For physical activity, studies [9,12,13,16,19,24,26] presenting data on moderate and high levels of practice separately, were brought together in a single group. In relation to the cut-off points for physical activity, the classifications used by each study were considered, which varied according to the measuring instrument used in their designs. For sedentary behavior, which was characterized in all studies by the screen time, a cut-off point ≤ 2 hours/day was adopted. This is because in three studies [10,13,14] screen time was considered low when ≤ 2 hours/day; in two other studies [12,15], < 2 hours/day; and in a single study [21] ≤ 16 hours/week (about 2 hours and 17min/day). For cardiorespiratory fitness, in studies [16,24,27] which presented moderate and high fitness data categories separately, these were brought together in a single group. Regarding the cut-off points, the classifications adopted by each study were considered, primarily distribution tertiles [16,24,27] or low vs. high fitness [8,23].

#### Analysis of sensitivity and subgroups

Subsequently, in order to verify whether studies with a greater risk of bias could be affecting the results of the meta-analysis, sensitivity analysis was performed, in which studies of low methodological quality were excluded. Several subgroup analyses were also performed, to identify other variables which could influence the results of the primary analysis. Regarding physical activity, the two forms of measurement (questionnaire and accelerometer) were considered separately. For sedentary behavior, the studies that measured the screen time for every day of the week and those that only considered the weekend were considered separately. In the case of cardiorespiratory fitness, the various measurement protocols were considered separately (field tests or laboratory tests, submaximal or maximal). Additional subgroup analyses were also performed involving: a) crude ORs; b) ORs adjusted for confounding variables; c) high prevalence of MetS (> 6.7% [prevalence above the median in relation to the studies included in the meta-analysis]); d) low prevalence of MetS (≤ 6.7% [prevalence at or below the median in relation to the studies included in the meta-analysis]); e) each diagnostic criteria for MetS.

## Results

### Qualitative synthesis of the studies

**Fig 1:**
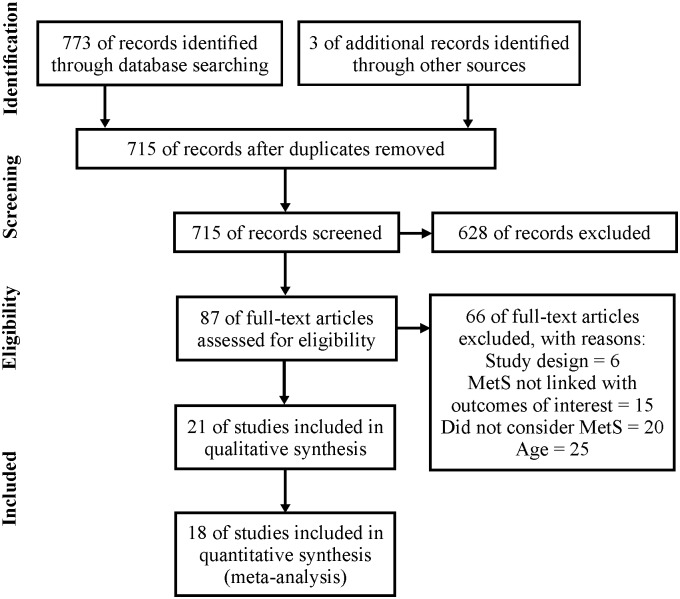
PRISMA flowchart presenting the summary of searches carried out in the literature.

In the databases considered, 773 studies were located. In addition, the manual search enabled the addition of three more studies. After exclusion of duplicate studies in the databases, an initial total of 715 studies was considered. Subsequently, titles and abstracts were analyzed and 628 studies were excluded, leaving 87 studies for analysis of the text in full. After reading the full texts, 66 studies did not meet the eligibility criteria (a complete list of studies excluded at this stage is available in S2 Table). The grounds for exclusion were: study design (6 studies); MetS not linked with outcomes of interest (15 studies); did not consider MetS (20 studies); and age (25 studies). Thus, 21 studies [7–27] were included in the qualitative synthesis and of these, 18 [8–10,12–21,23–27] offered sufficient data for inclusion in the meta-analysis (Fig 1).

### Methodological quality of studies

Scores attributed to the methodological quality of the studies, located and selected for analysis by systematic review procedures, presented amplitude of variation between 4 and 10 points (Tables 1, 2 and 3), and a mean score equivalent to 7.8 ± 1.5 points. Taking into consideration all the studies included in the systematic review, 10 achieved scores ≥ 9 (high quality) [11,13–16,19,21–24], and nine studies scored 6–8 (moderate quality) [7–9,12,18,20,25–27]. Only two studies attained scores between 4–5 (low quality) [10,17]; and no study achieved a score ≤ 3 (very low quality). S3 Table details the scores assigned to each study.

**Table 1:** Studies associating physical activity with metabolic syndrome in adolescents.

Study, country	Sample (number, gender, age)	Design	MetS (criteria and prevalence)	Physical activity measure and classification criteria	Adjustments	Results (*p* < 0.05)	Qual 0–10
Bermúdez-Cardona [7] (2016), Colombia	N = 96, 54 (M), 42 (F), 10–18 yrs	CC	Ford [44], MetS = 32 (obese group with MetS)	Questionnaire (3 DPAR): METs (day); MVPA (blocks); VPA (blocks)	Not applicable	No difference between groups (obese with MetS, obese without MetS and normal weight without MetS)	8
Rafraf [9] (2014), Iran	N = 216, 216 (F), 14–17 yrs	CS	De Ferranti [45], MetS = 10.6%	Questionnaire (IPAQ): METs (< 3 = low; 3–6 = moderate; ≥ 6 = vigorous)	Not applicable	No difference between groups with and without MetS	8
Fadzlina [10] (2014), Malaysia	N = 1,014, 387 (M), 627 (F), 13 yrs	CS	IDF [48], MetS = 2.6%	Questionnaire: low or moderate/high	NR	No association	4
Fam [11] (2013), Iran	N = 777, 457 (M), 320 (F), 12–18 yrs	CS	Cook [46], MetS = 13.8%	Questionnaire (MAQ): METs (< 3 = low; 3–6 = moderate; ≥ 6 = vigorous)	Age, gender and maternal education	No association	9
Mehairi [12] (2013), Arab Emirates	N = 998, 515 (M), 483 (F), 12–18 yrs	CS	IDF [48], MetS = 13%	Questionnaire (IPAQ): METs (< 3 = low; 3–6 = moderate; ≥ 6 = vigorous)	No adjustment	No association	6
Múnera [13] (2012), Colombia	N = 225 (overweight or obese), 115 (M), 110 (F), 10–18 yrs	CS	Harmonized criteria*, MetS = 3.1%	Questionnaire (3 DPAR): METs (day); VPA (≥ 1 block of 30 min); VPA (≥ 2 blocks of 30 min)	Not applicable	No difference between groups (no components, with one, two, or three components of MetS)	9
You [14] (2012), Korea	N = 606, 331 (M), 275 (F), 12–18 yrs	CS	Cook [46], MetS = 13%	Questionnaire (12 months): Number of days of VPA in the week (≤ 3 or > 3)	NR	Higher prevalence of MetS among those with higher VPA. No association	9
Tavares [15] (2012), Brazil	N = 210, 100 (M), 110 (F), 12–19 yrs	CS	Harmonized criteria*, MetS = 6.7%	Questionnaire (15 days): active or underactive	Not applicable	No difference in the prevalence of MetS between PA levels	9
Stabelini Neto [16] (2011), Brazil	N = 456, 233 (M), 223 (F), 10–18 yrs	CS	Cook [46], MetS = 7.7%	Questionnaire (3 days): MVPA min/day (< 60 = inactive; ≥ 60 and < 90 = active; ≥ 90 = very active)	Age and gender	Greater prevalence of MetS according to the decrease in PA. No association	9
Mikołajczak [17] (2011), Poland	N = 778, 369 (M), 409 (F), 16–18 yrs	CS	Harmonized criteria*, MetS = 7.1%	Questionnaire: low or moderate/high	NR	No association	5
Aboul Ella [18] (2010), Egypt	N = 4,252, 1,807 (M), 2,445 (F), 10–18 yrs	CS	Harmonized criteria*, MetS = 7.4%	Questionnaire (7 days): active or inactive	NR	OR 1.9 [CI 95%, 1.5 to 2.47] for inactive and MetS	7
Nguyen [19] (2010), Vietnam	N = 495, NR (M), NR (F), 13–16 yrs	CS	IDF [48], MetS = 4.6%	Accelerometer (7 days): median MVPA (≥ 3 METs); min/day and quartiles	Age and economic situation	Median MVPA was lower in the group with MetS. OR 5.3 [CI95%, 1.5 to 19.1] for lower PA and MetS (1^st^ vs. 4^th^ quartile)	9
Budak [20] (2010), Turkey	N = 790, 349 (M), 441 (F), 12–19 yrs	CS	Harmonized criteria*, MetS = 10.8%	Questionnaire: low or moderate/high	No adjustment	No association	8
Ekelund [23] (2009), Denmark, Estonia and Portugal	N = 1,535, NR (M), NR (F), 10–15 yrs	CS	IDF [48], MetS = 0.8%	Accelerometer (4 days): total time of daily PA	Age, gender and nationality	No difference for PA between the groups with or without MetS. OR 0.4 [CI 95%, 0.18 to 0.88] for longer daily PA and lower chance of MetS	9
McMurray [24] (2008), USA	N = 389, 212 (M), 177 (F), 14–17 yrs (at follow-up)	PRO (7 yrs)	Jolliffe [47], MetS = 4.6% (at follow-up)	Questionnaire (YHS): PA (total) score and tertiles (low, moderate, high)	Gender, BMI, blood pressure and cholesterol	MetS group (14–17 yrs) presented lower PA (baseline and follow-up). OR 5.11 [CI 95%, 1.05 to 49.13] for low (vs. High) PA in childhood (7–10 yrs) and MetS in adolescence	9
Mark [25] (2008), USA	N = 1,803, 1,005 (M), 798 (F), 12–19 yrs	CS	Jolliffe [47], MetS = 5.9%	Questionnaire (30 days): MVPA min/day (0–14; 15–29; 30–44; ≥ 45)	Age, smoking and screen time	No association	8
Pan [26] (2008), USA	N = 3,457, NR (M), NR (F), 12–19 yrs	CS	Harmonized criteria*, MetS = 3.5%	Questionnaire (30 days): average MVPA in min/day (< 30th percentile = low; 30–60 = moderate; ≥ 60 = high)	Not applicable	No difference in the prevalence of MetS between PA levels	7

M: male; F: female; NR: not reported; CC: case-control study; CS: cross-sectional study; PRO: prospective study; IDF: International Diabetes Federation; MetS: Metabolic syndrome; PA: Physical activity; METs: Metabolic equivalent; MVPA: moderate-to-vigorous physical activity; VPA: vigorous physical activity; 3DPAR: 3-day Physical Activity Recall; IPAQ: International Physical Activity Questionnaire; MAQ: modifiable activity questionnaire; YHS: Youth Health Survey; BMI: body mass index; OR: odds ratio; CI 95%: Confidence interval of 95%;

*Harmonized criteria refers to the use in combination of two or more criteria.

**Table 2:** Studies associating sedentary behavior with metabolic syndrome in adolescents.

Study, country	Sample (number, gender, age)	Design	MetS (criteria and prevalence)	Sedentary behavior measure and classification criteria	Adjustments	Results (*p* < 0.05)	Qual 0–10
Bermúdez-Cardona [7] (2016), Colombia	N = 96, 54 (M), 42 (F), 10–18 yrs	CC	Ford [44], MetS = 32 (obese group with MetS)	Recall (3 DPAR): screen hours/day (TV)	Not applicable	No difference between groups (obese with MetS, obese without MetS and normal weight without MetS)	8
Fadzlina [10] (2014), Malaysia	N = 1,014, 387 (M), 627 (F), 13 yrs	CS	IDF [48], MetS = 2.6%	Questionnaire (weekdays and weekend): Screen hours/day (≤ 2 or > 2)	NR	No association	4
Mehairi [12] (2013), Arab Emirates	N = 993, 509 (M), 484 (F), 12–18 yrs	CS	IDF [48], MetS = 13%	Questionnaire (7 days): Screen hours/day (< 2 or ≥ 2)	Age and type of school	No association	6
Múnera [13] (2012), Colombia	N = 225 (overweight or obese), 115 (M), 110 (F), 10–18 yrs	CS	Harmonized criteria*, MetS = 3.1%	Recall (3 DPAR): screen hours/day (TV)	Not applicable	No difference between groups (no components, with one, two, or three components of MetS)	9
You [14] (2012), Korea	N = 606, 331 (M), 275 (F), 12–18 yrs	CS	Cook [46], MetS = 13%	Questionnaire (one weekend): screen hours/day (TV or COM; ≤ 2 or > 2)	NR	Higher prevalence of MetS among those with longer TV, but not COM. OR 2.00 [CI 95%, 1.00 to 3.97] for greater TV time and MetS	9
Tavares [15] (2012), Brazil	N = 210, 100 (M), 110 (F), 12–19 yrs	CS	Harmonized criteria*, MetS = 6.7%	Questionnaire: Screen hours/day (< 2 or ≥ 2)	Not applicable	No difference in the prevalence of MetS between higher or lower screen time	9
Kang [21] (2010), Korea	N = 845, 449 (M), 396 (F), 10–18 yrs	CS	Ford [44], MetS = 7.3%	Questionnaire (7 days): Screen hours / week (TV and COM); total and quartiles (≤ 16; 17–24; 25–34; ≥ 35)	Age, gender, economic status and area of residence	Higher prevalence of MetS according to higher screen time. Screen time higher in the group with MetS. OR 2.23 [CI 95%, 1.02 to 4.86] for more screen time (≥ 35 h vs. ≤ 16 h) and MetS	10
Ekelund [23] (2009), Denmark, Estonia and Portugal	N = 2,900, NR (M), NR (F), 10–15 yrs	CS	IDF [48], MetS = 0.8%	Questionnaire: Screen hours/day (TV)	Not applicable	Screen time higher in the group with MetS	9
Mark [25] (2008), USA	N = 1,803, 1,005 (M), 798 (F), 12–19 yrs	CS	Jolliffe [47], MetS = 5.9%	Questionnaire (30 days): Screen hours/day (≤ 1, 2, 3, 4, or ≥ 5)	Age, smoking and physical activity	Higher prevalence of MetS according to higher screen time. OR 2.90 [CI 95%, 1.39 to 6.02] for higher screen time (≥ 5 h vs. ≤ 1 h) and MetS	8

M: male; F: female; NR: not reported; CC: case-control study; CS: cross-sectional study; IDF: International Diabetes Federation; MetS: Metabolic syndrome; 3DPAR: 3-day Physical Activity Recall; COM: computer; OR: odds ratio; CI 95%: Confidence interval of 95%;

*Harmonized criteria refers to the use in combination of two or more criteria.

**Table 3:** Studies associating cardiorespiratory fitness with metabolic syndrome in adolescents.

Study, country	Sample (number, gender, age)	Design	MetS (criteria and prevalence)	Cardiorespiratory fitness measure and classification criteria	Adjustments	Results (*p* < 0.05)	Qual 0–10
Laurson [8] (2015), Hungary	N = 379, 213 (M), 166 (F), 12–18 yrs	CS	Harmonized criteria*, MetS = 6.7%	VO_2Peak_ (maximal treadmill test): FitnessGram classification (“Healthy Fitness Zone "; "Need to improve"; or "Needs Improvement/Risk Zone”)	No adjustment	OR 3.9 [CI 95%, 1.6–9.1] for "Needs Improvement" and MetS or OR 4.7 [CI 95%, 2.0 to 11.0] for "Needs Improvement/Risk Zone" and MetS	6
Stabelini Neto [16] (2011), Brazil	N = 456, 233 (M), 223 (F), 10–18 yrs	CS	Cook [46], MetS = 7.7%	VO_2Max_ (20 meter shuttle run test): tertiles (low, moderate and high)	Age and gender	Greater prevalence of MetS according to the decrease in CRF. OR 3.0 [CI 95%, 1.13 to 7.94] for low CRF (vs. high) and MetS	9
Moreira [22] (2010), Portugal	N = 517, 220 (M), 297 (F), 15–18 yrs	CS	IDF [48], MetS = 5%	PACER (20 meters): FitnessGram classification (“below the healthy zone "; or "inside/above the healthy zone”)	Not applicable	Higher prevalence of MetS for those below the healthy zone	9
Ekelund [23] (2009), Denmark, Estonia and Portugal	N = 2,446, NR (M), NR (F), 10–15 yrs	CS	IDF [48], MetS = 0.8%	Maximal cycle ergometer test: watts per fat-free mass, per minute	Age, gender and nationality	Group with MetS presented lower values of CRF. OR 0.43 [CI 95%, 0.24 to 0.80] for high CRF and lower chance of MetS	9
McMurray [24] (2008), USA	N = 389, 212 (M), 177 (F), 14–17 yrs (at follow-up)	PRO (7 yrs)	Jolliffe [47], MetS = 4.6% (at follow-up)	VO_2Max_ (submaximal test on cycle ergometer): absolute and/or tertile value (low, moderate or high)	Gender, BMI, blood pressure and cholesterol level	MetS group (14–17 yrs) presented lower CRF (baseline and follow-up). OR 6.09 [CI 95%, 1.18 to 60.29] for low (vs. high) and OR 5.58 [CI 95%, 1.15 to 53.75] (vs. moderate) CRF in childhood (7–10 yrs) and MetS in adolescence	9
Janssen [27] (2007), USA	N = 1,561, 829 (M), 732 (F), 12–19 yrs	CS	Jolliffe [47], MetS = 7.6%	VO_2Max_ (submaximal treadmill test): tertiles (low, moderate or high)	Age, gender, ethnicity, smoking, economic status, lipid and carbohydrate intake	OR 0.18 [CI 95%, 0.07 to 0.48] for moderate and OR 0.01 [CI 95%, 0.00 to 0.07] for high CRF (vs. low) and lower chance of MetS	7

M: male; F: female; NR: not reported; CS: cross-sectional study; PRO: prospective study; IDF: International Diabetes Federation; MetS: Metabolic syndrome; CRF: cardiorespiratory fitness; VO_2Peak_: peak oxygen uptake; VO_2Max_: maximum oxygen consumption; PACER: Progressive aerobic cardiovascular and endurance run; BMI: body mass index; OR: odds ratio; CI 95%: Confidence interval of 95%;

*Harmonized criteria refers to the use in combination of two or more criteria.

### Physical activity

The 17 observational studies included in the systematic review which associated outcomes involving physical activity and MetS (Table 1 [7,9–20,23–26]) were published between 2008 and 2016. Of these, seven studies were from Asia [9–12,14,19,20], four from South America [7,13,15,16], three from North America [24–26], two from Europe [17,23] and one from Africa [18]. The total number of participants was 18,097 adolescents (ranging from 96 [7] to 4,252 [18]). Only one study did not include teenagers of both sexes (only girls) [9], and another included only overweight and obese adolescents [13]. Only one case-control study was included [7] and one prospective cohort [24], all other studies had a cross-sectional design [9–20,23,25,26]. MetS was diagnosed primarily through harmonized criteria (six studies [13,15,17,18,20,26]). Four studies used the criteria of the IDF [10,12,19,23], while the remainder used different criteria adapted for adolescents, derived from the NCEP-ATPIII [7,9,11,14,16,24,25]. MetS prevalence ranged from 0.8% [23] to 13.8% [11] (except for the case-control study [7], in which a sample of 32 adolescents with MetS had been previously selected). Details on the occurrence of MetS events in different classifications of physical activity are presented in S4 Table. The main resource used to monitor physical activity was questionnaires [7,9–12–14,15–17,18,20,24–26]. Two studies used accelerometers [19,23].

Regarding the results reported in the selected studies comparing the level of physical activity among adolescent carriers and non-carriers of MetS, four studies found no differences [7,9,13,23]; and two identified less physical activity in the MetS carrier adolescent group [19,24]. In studies in which the prevalence of MetS was compared among adolescents classified into different levels of physical activity, two studies showed no significant differences [15,26], one study found a higher prevalence of MetS according to less physical activity [16] and another study, in contrast, showed a higher prevalence of MetS among adolescents with higher physical activity [14]. Regarding the association between physical activity and MetS, eight studies presented no statistically significant OR values [10–12,14,16,17,20,25]. Two studies indicated that adolescents with low levels of physical activity presented a two to five times increase in the chances of having MetS [18,19]; while another study found that high levels of physical activity offered a protective effect related to the appearance of MetS (OR = 0.4 [CI 95%, 0.18 to 0.88]) [23]. One prospective study found that low levels of physical activity in childhood increased by about five times the odds of the presence of MetS in adolescence [24]. In relation to the adjustments for confounding variables, two studies ignored this procedure [12,20]; and four studies demonstrated adjusted OR values, but did not report the variables selected in the adjustments [10,14,17,18]. The other studies performed adjustments for age and/or gender [11,16,19,23–25]. Only one study adjusted for body mass index [24] economic situation [19], sedentary behavior and smoking [25].

### Sedentary behavior

For the nine studies that associated outcomes involving sedentary behavior and MetS (Table 2 [7,10,12–15,21,23,25]), the year of publication ranged from 2008 to 2016. Of these, four studies were from Asia [10,12,14,21], three from South America [7,13,15], one from Europe [23] and one from North America [25]. The total number of participants was equal to 8,680 (ranging from 96 [7] to 2,900 adolescents [23]). One study included only overweight and obese adolescents [13]. Only one case-control study was included [7] and no cohort studies. Other studies presented a cross-sectional design [10,12–15,21,23,25]. MetS was primarily diagnosed by IDF criteria (3 studies [10,12,23]). Two studies used harmonized criteria [13,15], while the remainder used different criteria adapted for adolescents, from the NCEP-ATPIII [7,14,21,25]. The prevalence of MetS ranged from 0.8% [23] to 13% [12,14] (except for the case-control study [7], in which a sample of 32 adolescents with MetS had been previously selected). Details on the occurrence of MetS events in different classifications of sedentary behavior are presented in S5 Table. Sedentary behavior was treated by screen time in all studies, primarily identified by questionnaire [10,12,14,15,21,23,25]. Two studies used recall [7,13]. Three studies investigated only TV time [7,13,23]; two studies TV time and recreational computer use [14,21]; and the remaining four studies reported any form of screen-based entertainment [10,12,15,25].

Regarding the results reported in the literature, of the studies that identified differences in screen time among adolescent carriers and non-carriers of MetS, two showed no significant differences [7,13]; and two other studies found that screen time was higher in the group with MetS [21,23]. In studies that established comparisons between the prevalence of MetS and different screen times, one study identified no significant differences (< 2 hours/day vs. ≥ 2 hours/day) [15]; another study found a higher prevalence of MetS according to increased screen time, identified by distribution quartiles [21]; and a third study found a higher prevalence of MetS among adolescents who reported more TV time, however, this was not the case for recreational computer use (≤ 2 hours/day vs. > 2 hours/day) [14]. In another study, the prevalence of MetS increased with higher screen time (≤ 1, 2, 3, 4 ≥ 5 hours/day). Regarding the association between MetS and screen time, two studies found no statistical significance [10,12]. One study observed that higher TV time at weekends (> 2 hours/day) could double the chances of developing MetS [14]. Another study found that adolescents with screen time ≥ 35 hours/week tended to be twice as likely to develop MetS compared to their peers with screen time ≤ 16 hours/week [21]. Another study identified that screen time ≥ 5 hours/day increases the odds of developing MetS by about three times compared with screen time ≤ 1 hour/day [25]. Regarding adjustments for confounding factors, two studies demonstrated an adjusted OR, but did not report the variables selected for adjustment [10,13]. Other studies established adjustments mainly for age [12,21,25]. Only one study adjusted for smoking and physical activity [25], or gender, economic status and area of residence [21].

### Cardiorespiratory fitness

The six studies that associated outcomes involving cardiorespiratory fitness and MetS (Table 3 [8,16,22–24,27]) were published between 2007 and 2015. Of these, three studies were from Europe [8,22,23], two were conducted in North America [24,27] and one in South America [16]. In total, they included 5,748 participants, ranging from 379 [8] to 2,446 adolescents [23]. No case-control studies were found in the literature and only one prospective cohort study [24]; the other studies had cross-sectional designs [8,16,22,23,27]. MetS was diagnosed in two studies by means of the criteria proposed by the IDF [22,23] and one study by harmonized criteria [8], the other three studies used criteria derived from the NCEP-ATPIII, adapted for teenagers [16,24,27]. The prevalence of MetS ranged from 0.8% [23] to 7.7% [16]. Details on the occurrence of MetS events in different classifications of cardiorespiratory fitness are presented in S6 Table. To establish estimates of cardiorespiratory fitness, two studies used similar field tests (20-meter shuttle run test and endurance run test—PACER) [16,22], while the other four studies made use of a treadmill or cycle ergometer through maximal [8,23] and submaximal load tests [24,27].

Regarding the results reported in publications, the studies comparing scores equivalent to VO_2_max, among adolescent carriers and non-carriers of MetS, identified lower cardiorespiratory fitness in adolescents with MetS [23,24]. Studies comparing the prevalence of MetS between different levels of cardiorespiratory fitness found higher prevalence, according to lower levels of cardiorespiratory fitness [16,22]. For measures of association between cardiorespiratory fitness and MetS, two studies demonstrated that higher cardiorespiratory fitness may offer a significant protective effect for MetS (OR = 0.43 [CI 95%, 0.18 to 0.88] and OR = 0.01 [CI 95%, 0.00 to 0.07]) [23,27]. Similarly, two other studies demonstrated that low cardiorespiratory fitness increased the risk of the onset and development of MetS by three to five times [8,16]. The one prospective cohort study pointed out that low cardiorespiratory fitness in childhood may increase by six times the chances of identifying MetS in adolescence [24]. Regarding adjustments for confounding variables, one study chose not to perform this procedure [8], while the remaining studies adjusted primarily for age and/or gender [16,23,24,27]. Furthermore, one study adjusted for body mass index [24] and another for ethnicity, smoking, economic status, and intake of lipids and carbohydrates [27].

### Quantitative synthesis of studies (meta-analysis)

#### Primary analysis and sensitivity

**Fig 2:**
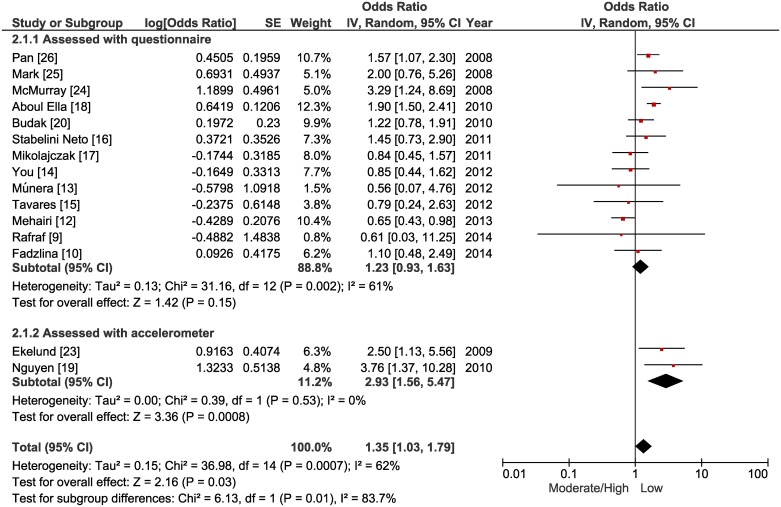
Forest plot of the primary and subgroup analysis comparing odds ratios for metabolic syndrome among adolescents with moderate/high levels of physical activity versus low level of physical activity.

**Fig 3:**
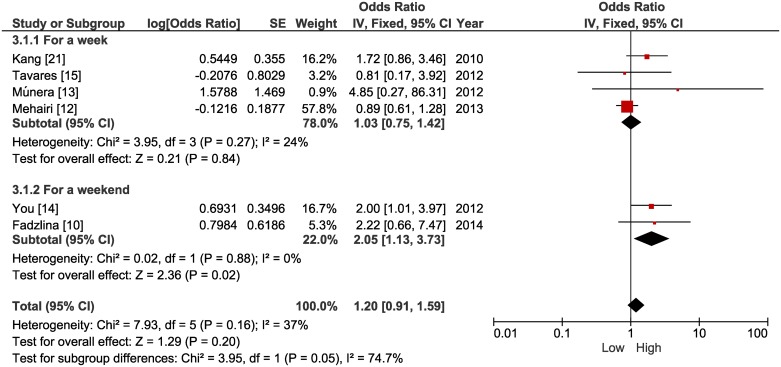
Forest plot of the primary and subgroup analysis comparing odds ratios for metabolic syndrome among adolescents with low screen time versus high screen time.

**Fig 4:**
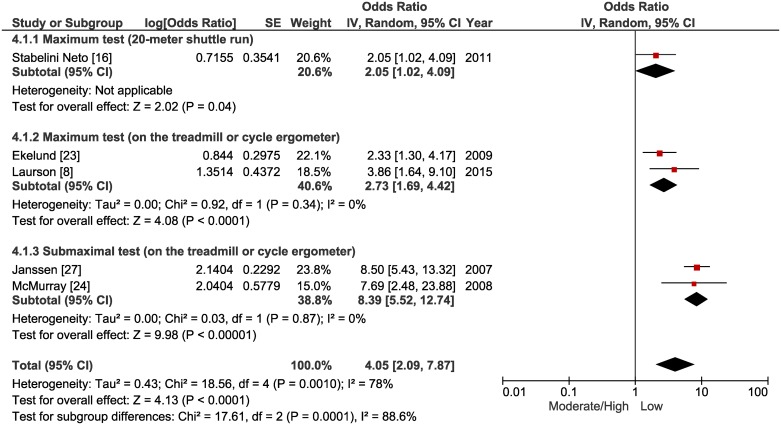
Forest plot of the primary and subgroup analysis comparing odds ratios for metabolic syndrome among adolescents with moderate/high cardiorespiratory fitness versus low cardiorespiratory fitness.

In the primary analysis a significant association was identified between low levels of physical activity and a higher chance of developing MetS (OR = 1.35 [CI 95%, 1.03 to 1.79] p = 0.03, n = 17,224, studies = 15, I^2^ = 62%; Fig 2). In relation to sedentary behavior, a significant association was not identified between screen time and MetS (OR = 1.20 [CI 95%, 0.91 to 1.59] p = 0.20, n = 3,881, studies = 6, I^2^ = 37%; Fig 3), while low cardiorespiratory fitness was significantly associated with a higher chance of developing MetS (OR = 4.05 [CI 95%, 2.09 to 7.87] p < 0.0001, n = 5,231, studies = 5, I^2^ = 78%; Fig 4).

For the sensitivity analysis, studies were excluded that addressed the association between MetS and physical activity [10,17] or sedentary behavior [10], due to low methodological quality. In the case of cardiorespiratory fitness, even considering that no study was categorized as low methodological quality, one study was excluded [8] due to possible increased risk of bias, for presenting lower scores equivalent to methodological quality. However, results found in the primary analysis showed no alterations after exclusion of these studies (S1, S2 and S3 Figs).

#### Subgroup analyses

For physical activity, two subgroup analyses were performed (Fig 2), based on the different measuring instruments used in the studies. When considering the studies that used questionnaires (OR = 1.23 [CI 95%, 0.93 to 1.63] p = 0.15, n = 15,194, studies = 13, I^2^ = 61%), no significant association was observed between physical activity and MetS. However, when measured through the use of accelerometers, a low level of physical activity was significantly associated with a higher chance of developing MetS (OR = 2.93 [CI 95%, 1.56 to 5.47] p = 0.0008, n = 2,030, studies = 2, I^2^ = 0%).

Regarding sedentary behavior, two subgroup analyses were carried out (Fig 3). In the first, studies were considered that measured screen time all week. In this case, there was no significant association between screen time and MetS (OR = 1.03 [CI 95%, 0.75 to 1.42] p = 0.84, n = 2,261, studies = 4, I^2^ = 24%). However, when taking into account screen time specifically at the weekend there was a significant association (OR = 2.05 [CI 95%, 1.13 to 3.73] p = 0.02, n = 1,620, studies = 2, I^2^ = 0%), between higher screen time and an increased chance of developing MetS.

For cardiorespiratory fitness, analyses were grouped into three types of tests to estimate VO_2_max (Fig 4). When considering studies that used maximal field tests (20-meter shuttle run test) (OR = 2.05 [CI 95%, 1.02 to 4.09] p = 0.04, n = 456, studies = 1) and maximal (OR = 2.73 [CI 95%, 1.69 to 4.42] p < 0.0001, n = 2,825, studies = 2, I^2^ = 0%) or submaximal load tests (OR = 8.39 [CI 95%, 5.52 to 12.74] p < 0.00001, n = 1.950, studies = 2, I^2^ = 0%) on a treadmill or cycle ergometer, significant associations were observed between low cardiorespiratory fitness and a higher chance of developing MetS.

Additional analyses of subgroups were performed for: crude ORs (S4, S5 and S6 Figs) vs. Adjusted ORs (S7, S8 and S9 Figs), studies with a high prevalence of MetS vs. studies with a low prevalence (S10, S11 and S12 Figs), and according to the diagnostic criteria used (S13, S14 and S15 Figs). When crude OR data were selected (without adjustments for confounding variables), physical activity was not significantly associated with MetS, either through the use of a questionnaire or the accelerometry technique (S4 Fig). High sedentary behavior on the weekend was associated with the development of MetS (OR = 1.94 [CI 95%, 1.23 to 3.07] p = 0.004, n = 1.620, studies = 2, I^2^ = 0%), but not when considering the whole week (S5 Fig), while low cardiorespiratory fitness was significantly associated with the development of MetS independent of the technique used (S6 Fig).

When grouping studies with adjusted OR data, low physical activity was significantly associated with MetS only when using accelerometry (OR = 2.50 [CI 95%, 1.13 to 5.56] p = 0.02, n = 2,446, studies = 1), not when using a questionnaire (S7 Fig). For sedentary behavior no significant association was found (S8 Fig), while low cardiorespiratory fitness was significantly associated with MetS (OR = 2.33 [CI 95%, 1.30 to 4.17] p = 0.005, n = 2,446, studies = 1; S9 Fig).

When only studies that found a high MetS prevalence were grouped, no significant association was found with physical activity, however, for studies that found a low MetS prevalence, a significant association was observed between low physical activity level and a higher risk of MetS (OR = 1.78 [CI 95%, 1.27 to 2.50] p = 0.0009, n = 9,128, studies = 8, I^2^ = 22%, S10 Fig). For sedentary behavior, no significant association was found considering studies with a high or low prevalence of MetS (S11 Fig). On the other hand, low cardiorespiratory fitness was associated with a higher risk of MetS when considering studies with a high (OR = 4.28 [CI 95%, 1.06 to 17.27] p = 0.04, n = 2,017, studies = 2, I^2^ = 91%) or low prevalence of MetS (OR = 3.57 [CI 95%, 1.88 to 6.80] p = 0.0001, n = 3.214, studies = 3, I^2^ = 45%, S12 Fig).

For the subgroup analysis in which each MetS diagnostic criterion was verified separately, a low level of physical activity was associated with a greater risk of developing MetS through the Jolliffe and Janssen [47] diagnostic criteria (OR = 2.56 [CI 95%, 1.29 to 5.08] p = 0.007, n = 2,192, studies = 2, I^2^ = 0%), or when using harmonized criteria (OR = 1.36 [CI 95%, 1.01 to 1.84] p = 0.04, n = 9,712, studies = 6, I^2^ = 49%). For the remaining diagnostic criteria, there were no significant associations between physical activity and MetS (S13 Fig). Regarding sedentary behavior, no significant association was observed when the studies were grouped by diagnostic criteria (S14 Fig). However, low cardiorespiratory fitness was significantly associated with the development of MetS, independent of the diagnostic criteria used (S15 Fig).

## Discussion

### Agreements and disagreements with other studies

To our knowledge, to date, no meta-analysis involving adolescents has investigated the variables selected in this study. A previously performed systematic review suggested evidence that low levels of physical activity may be associated with an increased risk of the onset and development of MetS in adolescents [31]. Primary and sensitivity analysis performed in the present study confirmed the existence of a significant association between physical activity and MetS. From the qualitative analyses of the studies included in the systematic review, it was not possible to predict this finding, since only four [18,19,23,24] of the 12 selected studies that sought to identify an association between physical activity and MetS found significant results.

Among the selected studies, only two used the accelerometry technique to measure physical activity [19,23], which provides more reliable results [33]. Nguyen et al. [19] found that being located in the lowest quartile of physical activity increased the chances of developing MetS by more than five times, and Ekelund et al. [23] identified a lower risk (OR = 0.4 [CI 95%, 0.18 to 0.88]) for the development of MetS in adolescents with higher levels of physical activity. These findings available in individual studies were confirmed in the subgroup analysis performed in the present study, in which a significant association between MetS and physical activity was not identified when evaluated by self reported measures (questionnaire), however, in the analysis that considered the two studies in which physical activity was scaled using accelerometers, it was found that low levels of physical activity increase the odds of developing MetS by approximately three times.

In adolescents the use of self reported measures of physical activity can increase the possibility of error, since this population tends to present more difficulty remembering and reporting accurately the intensity, frequency and duration of activities [33]. Previous studies indicate that self reported measures tend to overestimate the actual level of physical activity [51]. In addition, some questionnaires have not been sufficiently validated against objective methods, creating doubts about their effective ability to properly evaluate the level of physical activity, especially among young people [33].

Thus, it is possible to assume that the effect size of the association measure of the primary analysis for physical activity was underestimated. In principle, it was found that low levels of physical activity increase the chances of developing MetS by 35%. However, the subgroup analysis with measurement of physical activity using accelerometers demonstrated about a three times higher chance of developing MetS, suggesting that this could be the actual size of the effect if other studies including more precise measurements of physical activity had been available. However, the low number of studies in this subgroup analysis restricted safer extrapolations. It should also be considered that a significant association between physical activity and MetS observed using the accelerometry technique was dependent on OR adjusted for confounding variables (age, gender, and nationality), since when only crude ORs were considered, no significant association was observed.

The subgroup analyses also showed that a low level of physical activity was significantly associated with a 78% greater risk of developing MetS when grouping studies that found a low prevalence of the syndrome, whereas a significant association was not found in studies that observed a high prevalence. This may have been due to the fact that studies that find a high prevalence of MetS usually rely on less rigorous criteria to diagnose the syndrome. It has been shown that less rigorous criteria (e.g.: de Ferranti et al. [45]) can overestimate the proportion of adolescents with MetS [6]. This argument is reinforced by the subgroup analyses in which the studies were separated by diagnostic criteria. In this case, the association between low levels of physical activity and MetS was maintained only for the Jolliffe and Janssen criteria [47] and for studies using harmonized criteria, in which a low prevalence of MetS was found in the majority of studies [13,15,24,25,26]. The exception was the IDF criterion, which despite being rigorous to classify adolescents with MetS [6] presented no significant association in the subgroup analysis.

In relation to sedentary behavior, an important limitation was the use of self-reported measurements for screen time in all studies, possibly sacrificing the quality of the treated measure. Another fact that should be considered is the cut-off point adopted for the screen time in several studies [10,12–14,15], which typically referred to ≤ 2 hours/day. This cut-off point was defined by consensus among specialists who sought to establish guidelines based on evidence for health with respect to sedentary behavior in children and adolescents [52].

However, in the primary and sensitivity analyses performed based on this cut-off point, there were no significant associations between screen time and MetS. Individual studies included in the systematic review that showed significant associations used different cut-off points. Kang et al. [21] found that screen time equivalent to ≥ 35 hours/week (approximately ≥ 5 hours/day) compared to ≤ 16 hours/week (approximately ≤ 2 hours and 17 min/day) lead to two times the chances of developing MetS. Similarly, Mark and Janssen [25] only identified an association when considering screen time ≥ 5 hours/day versus ≤ 1 hour/day, indicating in this case close to three times more risk of developing MetS. The exception was the study of You and Son [14], who found twice the risk for MetS when screen time was > 2 hours/day, however, specifically on weekend days.

The subgroup analysis indicated that screen time > 2 hours/day on the weekend, revealed a two times greater chance of developing MetS in adolescents, while an association involving days of the week, showed no statistical significance. Kang et al. [21], when analyzing the association between screen time and MetS, considering only the weekend, identified a risk almost three times greater for MetS, but only for the screen time strata ≥ 7 hours/day versus ≤ 3 hours/day. In the same sample, the researchers found no association when considering only days of the week (excluding weekends). In particular, there seems to be evidence in the sense that there may be a greater association between screen time at weekends and MetS in adolescents, than during weekdays. However, the subgroup analysis showed that when using only OR adjusted for confounding variables, the significant association between screen time at weekends and MetS disappeared.

When only studies with a high or low prevalence of MetS were considered separately, no significant association was observed between screen time and MetS, in the same way as when studies were grouped by diagnostic criteria. In this case, the same limitations as previously presented should be considered (use of self-reported measures, cut-off point ≤ 2 hours/day, and low number of studies in the meta-analysis). In addition, the primary analysis had already indicated that there was no significant association between sedentary behavior and MetS.

When establishing associations between cardiorespiratory fitness and MetS in adolescents, the primary analysis indicated that lower VO_2_max values can present an approximately four times greater chance of MetS. Data analyzed qualitatively in the systematic review had already indicated this trend. The five selected individual studies [8,16,23,24,27] showed significant associations between cardiorespiratory fitness and MetS in adolescents.

The subgroup analyses demonstrated that the significant association is independent of the three different types of tests used. Despite having provided a smaller effect size (OR = 2.05 [CI 95%, 1.02 to 4.09]), the test used (20-meter shuttle run test) by Stabelini Neto [16], has the advantage of being administered in large numbers of subjects simultaneously, in a short time and at low cost. In addition, a previously performed meta-analysis demonstrated that VO_2_max estimated using the 20-meter shuttle run test presents good correlation (r = 0.78) with methods involving gas exchange in adolescents [53].

Given that maximal and submaximal tests involving cycle ergometers and treadmills may not be feasible in many places, the multi-stage 20-meter test constitutes a useful alternative to estimate cardiorespiratory fitness, especially for adolescents in a school environment [53]. This type of test can be used in an attempt to identify adolescents with low cardiorespiratory fitness and a consequently greater metabolic risk. Moreira et al. [22], using a similar test (PACER), found a significantly higher prevalence of MetS in the group of adolescents with low cardiorespiratory fitness (8.5%) compared with the group of adolescents with high cardiorespiratory fitness (0%). However, it was only possible to select one study involving this type of testing for the subgroup analysis, which could limit any generalizations.

Finally, the association between low cardiorespiratory fitness and MetS occurred regardless of the use of crude OR or adjusted for confounding variables. In addition, considering the strong association between these variables already presented in the primary analysis, it was observed that a significant association between low cardiorespiratory fitness and MetS remained independent of the prevalence found, or of the diagnostic criteria used.

### Quality of evidence

Of the 15 studies included in the meta-analysis involving physical activity, there was representation from five different geographical regions (Asia [9,10,12,14,19,20] Latin America [13,15,16], North America [24,25,26], Europe [17,23] and Africa [18]), while for sedentary behavior only two regions (Asia [10,12,14,21] and Latin America [13,15]) and for cardiorespiratory fitness, three regions (North America [24,27], Europe [8,23] and Latin America [16]). Thus, it is not possible to extrapolate the results to all locations, since there was no reasonable representation from the different regions. In contrast, the quality of the studies included in the systematic review was considered moderate, with a mean score of 7.8 ± 1.5 points. Only two studies [10,17] presented low quality indicators and sensitivity analysis demonstrated that the exclusion of both studies did not change the results of the primary analysis.

However, of the 18 studies included in the meta-analysis, only four [12,14,16,27] presented data separately for boys and girls, which made subgroup analysis impossible for this characteristic. Similarly, the studies did not present data for different ages in adolescence, preventing subgroup analyses for this factor. Furthermore, MetS was identified according to five different diagnostic criteria (IDF [10,12,19,22,23] Cook [11,14,16], Jolliffe and Janssen [24,25,27], Ford [7,21], and de Ferranti [9]), as well as some researchers [8,13,15,17,18,20,26] using harmonized criteria. This is configured as a limiting factor to be taken into account when interpreting the results, since the prevalence of MetS in adolescence can vary significantly when diagnosed by different criteria [6]. In addition, some studies did not adjust the measures of association taking into consideration possible confounding variables [8,12,20], while others, despite reporting the use of covariables, did not specify which ones were used [10,14,17,18].

### Potential biases in the review process

In the systematic review and meta-analysis only observational studies were included, which increases the risk of bias, especially since the majority of the studies selected were cross-sectional, thus hindering the performance of causal inferences. The number of studies available for meta-analysis should also be raised as another limitation. Only one analysis was carried out with ≥ 10 studies (primary analysis, the odds ratio for metabolic syndrome in adolescents with moderate/high levels of physical activity versus low levels of physical activity), thus allowing visualization on the funnel plot (S16 Fig), which showed no evidence of publication bias. Moreover, the majority of analyses used random effects models, considering the heterogeneity of the data, which reduces the effect of the associations and, therefore, the results should be interpreted with caution. Finally, the search did not extend to all existing databases. Nonetheless, we performed searches in three primary databases (PubMed, SPORTDiscus, and LILACS) and one secondary database (The Cochrane Library). In addition, we conducted a thorough search of all the references of the studies included in the review in an attempt to find further studies.

## Conclusions

### Implications for research

The study demonstrated that low levels of physical activity were significantly associated with the development of MetS in adolescents; however, the subgroup analyses indicated that a significant effect was dependent on the measuring instrument (accelerometer). The analysis involving the self reported measurements did not indicate significant association, suggesting that the effect size of the primary analyses may have been underestimated. Thus, due to the limitations of questionnaire use in adolescents, future studies should prioritize the use of objective measures, aiming to investigate the association between physical activity and MetS in this population. Other issues that should be considered in future studies are the importance of using adjustments for covariables, in addition to preferentially using more rigorous criteria to diagnose MetS.

As for sedentary behavior, there was no significant association between screen time > 2 hours/day and MetS in adolescents. Although the subgroup analysis indicated a significant association when taking into account only screen time over the weekend, this association disappeared when OR adjusted for covariables was used. In view of this, it is suggested that future studies investigate screen time separately for weekdays, weekends and throughout the week. They should also be encouraged to perform analysis using different cut-off points for screen time, not solely > 2 hours/day. In addition, whenever possible, objective measures should be employed, avoiding the limitations related to self reported measures.

Finally, in relation to cardiorespiratory fitness, analyses showed the existence of a significant association between low VO_2_max and MetS. However, the primary analysis included few studies, preventing visual inspection of the funnel plot, as a way to detect the existence of possible unpublished studies due to no significant results. In view of this, in an attempt to verify whether the findings of this systematic review and meta-analysis are sustained, it is suggested that further studies be carried out, in order to identify the association between cardiorespiratory fitness and MetS in adolescent populations.

### Implications for practice

Among the variables analyzed in this systematic review and meta-analysis, a low level of physical activity, screen time > 2 hours/day at weekends and low cardiorespiratory fitness were the indicators that demonstrated significant associations with MetS in adolescents. Thus, an increase in time spent in physical activity of moderate to high intensity and reduced screen time of ≤ 2 hours/day at weekends should be encouraged, especially in adolescents with low cardiorespiratory fitness who are exposed to increased metabolic risk.

## Supporting Information

S1 Fig Forest plot of the sensitivity analysis comparing odds ratios for metabolic syndrome among adolescents with moderate/high levels of physical activity versus low level of physical activity. (PNG)

S2 Fig Forest plot of the sensitivity analysis comparing odds ratios for metabolic syndrome among adolescents with low screen time versus high screen time. (PNG)

S3 Fig Forest plot of the sensitivity analysis comparing odds ratios for metabolic syndrome among adolescents with moderate/high cardiorespiratory fitness versus low cardiorespiratory fitness. (PNG)

S4 Fig Forest plot of the subgroup analysis comparing crude odds ratios for metabolic syndrome among adolescents with moderate/high levels of physical activity versus low levels of physical activity. (PNG)

S5 Fig Forest plot of the subgroup analysis comparing crude odds ratios for metabolic syndrome among adolescents with low screen time versus high screen time. (PNG)

S6 Fig Forest plot of the subgroup analysis comparing crude odds ratios for metabolic syndrome among adolescents with moderate/high cardiorespiratory fitness versus low cardiorespiratory fitness. (PNG)

S7 Fig Forest plot of the subgroup analysis comparing adjusted odds ratio for metabolic syndrome among adolescents with moderate/high levels of physical activity versus low levels of physical activity. (PNG)

S8 Fig Forest plot of the subgroup analysis comparing adjusted odds ratios for metabolic syndrome among adolescents with low screen time versus high screen time. (PNG)

S9 Fig Forest plot of the subgroup analysis comparing adjusted odds ratios for metabolic syndrome among adolescents with moderate/high cardiorespiratory fitness versus low cardiorespiratory fitness. (PNG)

S10 Fig Forest plot of the subgroup analysis (high prevalence of metabolic syndrome vs. low prevalence) comparing odds ratios for metabolic syndrome among adolescents with moderate/high levels of physical activity versus low levels of physical activity. (PNG)

S11 Fig Forest plot of the subgroup analysis (high prevalence of metabolic syndrome vs. low prevalence) comparing odds ratios for metabolic syndrome among adolescents with low screen time versus high screen time. (PNG)

S12 Fig Forest plot of the subgroup analysis (high prevalence of metabolic syndrome vs. low prevalence) comparing odds ratios for metabolic syndrome among adolescents with moderate/high cardiorespiratory fitness versus low cardiorespiratory fitness. (PNG)

S13 Fig Forest plot of the subgroup analysis (according to the criteria used to diagnose the metabolic syndrome) comparing odds ratios for metabolic syndrome among adolescents with moderate/high levels of physical activity versus low levels of physical activity. (PNG)

S14 Fig Forest plot of the subgroup analysis (according to the criteria used to diagnose the metabolic syndrome) comparing odds ratios for metabolic syndrome among adolescents with low screen time versus high screen time. (PNG)

S15 Fig Forest plot of the subgroup analysis (according to the criteria used to diagnose the metabolic syndrome) comparing odds ratios for metabolic syndrome among adolescents with moderate/high cardiorespiratory fitness versus low cardiorespiratory fitness. (PNG)

S16 Fig Funnel plot of the primary analysis comparing odds ratios for metabolic syndrome among adolescents with moderate/high levels of physical activity versus low level of physical activity. (PNG)

S1 Table PRISMA Checklist. (DOCX)

S2 Table Studies excluded after reading the full text. (DOCX)

S3 Table Methodological quality of the 21 studies included in the systematic review. (DOCX)

S4 Table Metabolic syndrome events in different classifications of physical activity. (DOCX)

S5 Table Metabolic syndrome events in different classifications of sedentary behavior. (DOCX)

S6 Table Metabolic syndrome events in different classifications of cardiorespiratory fitness. (DOCX)
