# Supplementary figures and images for: Physical Activity, Sedentary Behavior, Cardiorespiratory Fitness and Metabolic Syndrome in Adolescents: Systematic Review and Meta-Analysis of Observational Evidence
Source: PLoS One. 2016 Dec 20;11(12):e0168503. doi: 10.1371/journal.pone.0168503 (PMC5173371; doi:10.1371/journal.pone.0168503)

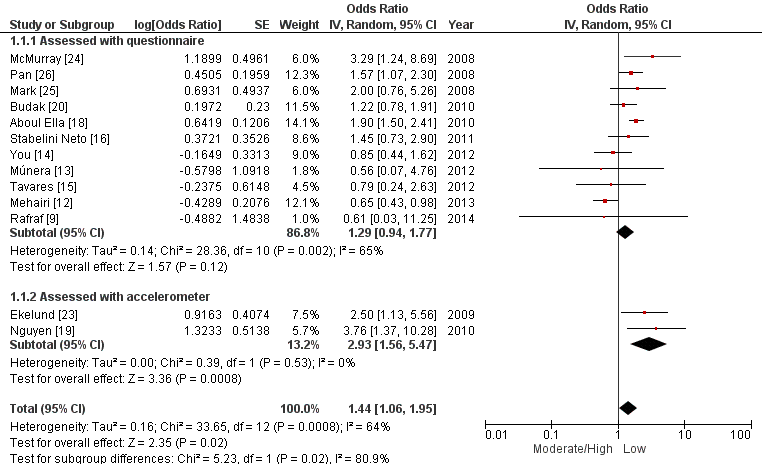

Supplement: S1 Fig — (PNG) [file pone.0168503.s001.png]

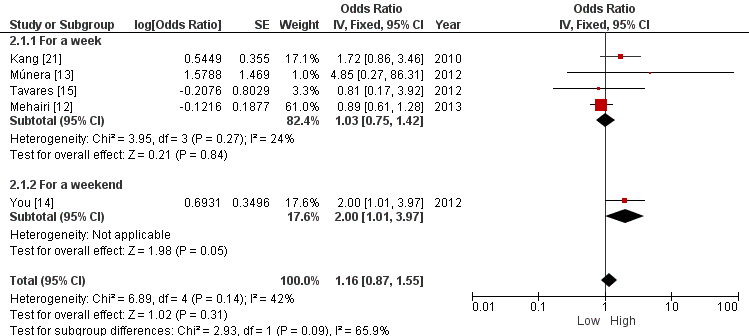

Supplement: S2 Fig — (PNG) [file pone.0168503.s002.png]

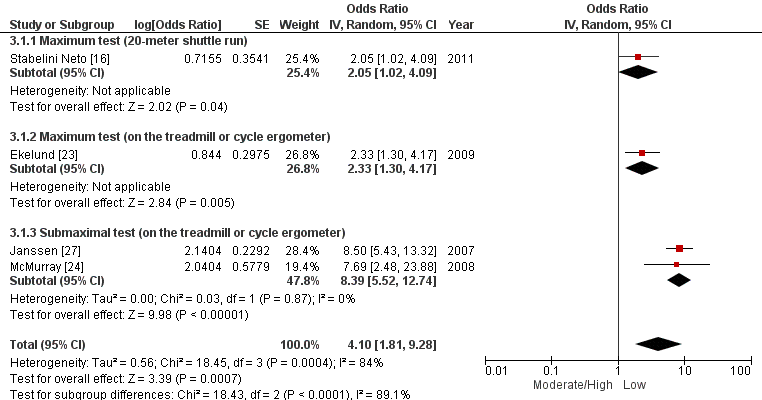

Supplement: S3 Fig — (PNG) [file pone.0168503.s003.png]

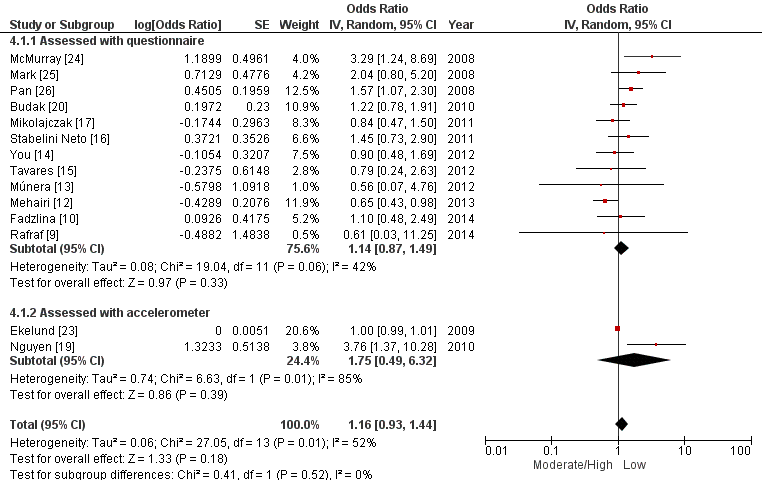

Supplement: S4 Fig — (PNG) [file pone.0168503.s004.png]

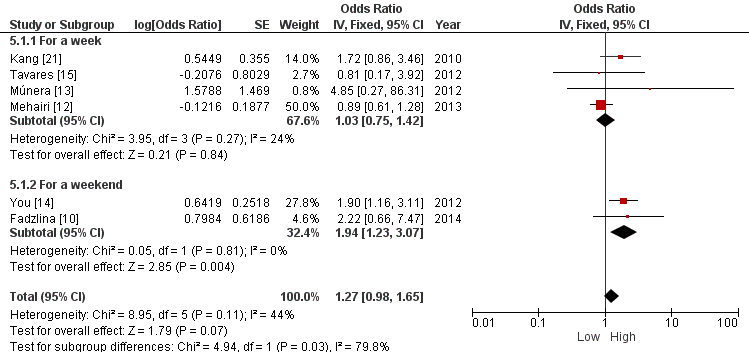

Supplement: S5 Fig — (PNG) [file pone.0168503.s005.png]

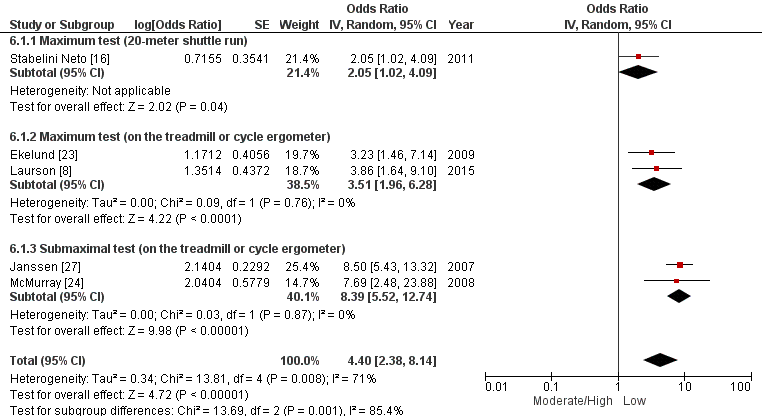

Supplement: S6 Fig — (PNG) [file pone.0168503.s006.png]

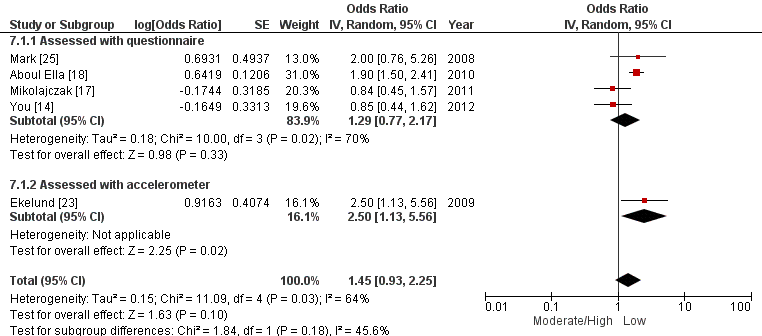

Supplement: S7 Fig — (PNG) [file pone.0168503.s007.png]

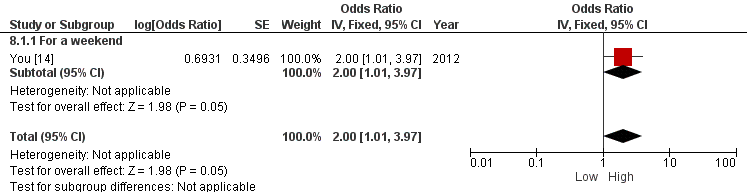

Supplement: S8 Fig — (PNG) [file pone.0168503.s008.png]

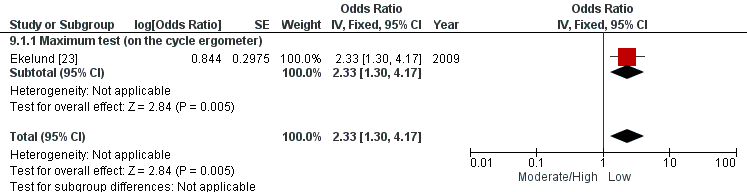

Supplement: S9 Fig — (PNG) [file pone.0168503.s009.png]

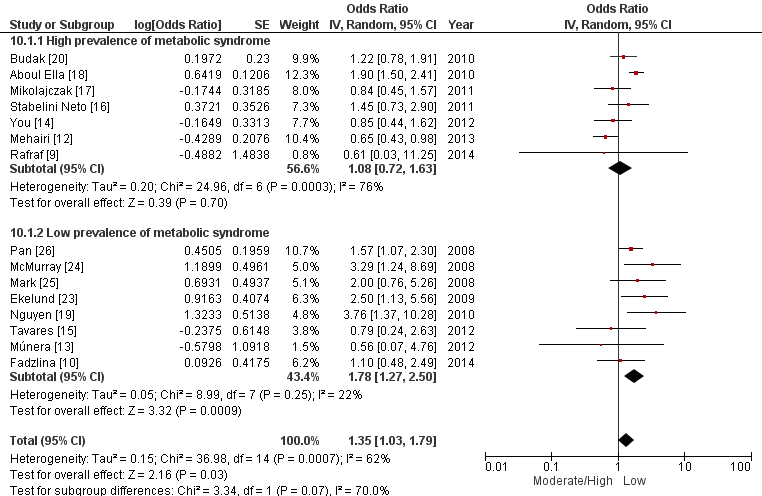

Supplement: S10 Fig — (PNG) [file pone.0168503.s010.png]

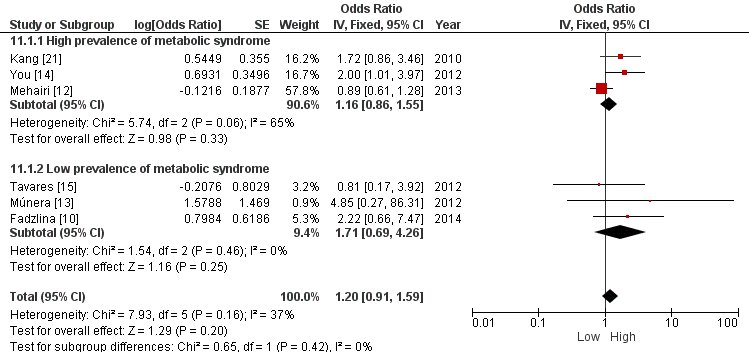

Supplement: S11 Fig — (PNG) [file pone.0168503.s011.png]

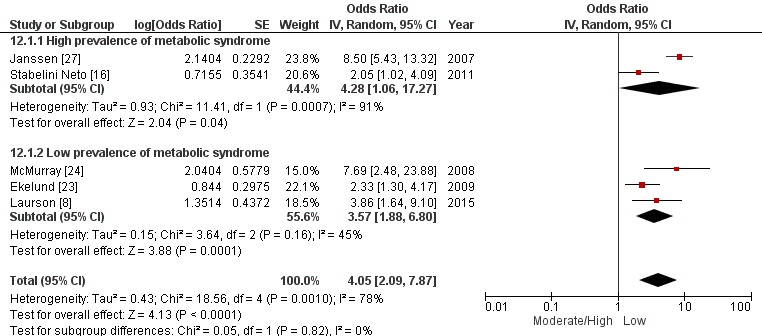

Supplement: S12 Fig — (PNG) [file pone.0168503.s012.png]

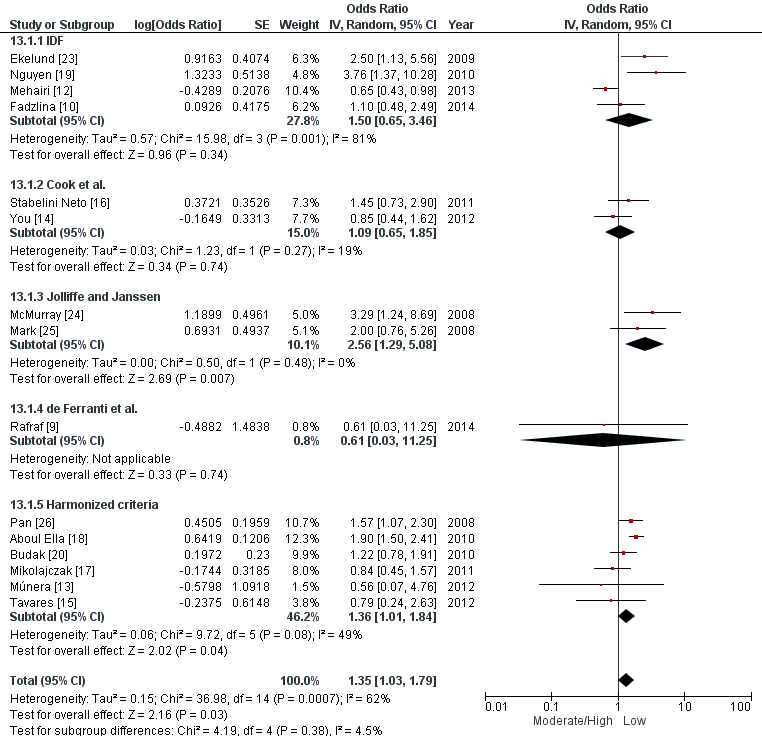

Supplement: S13 Fig — (PNG) [file pone.0168503.s013.png]

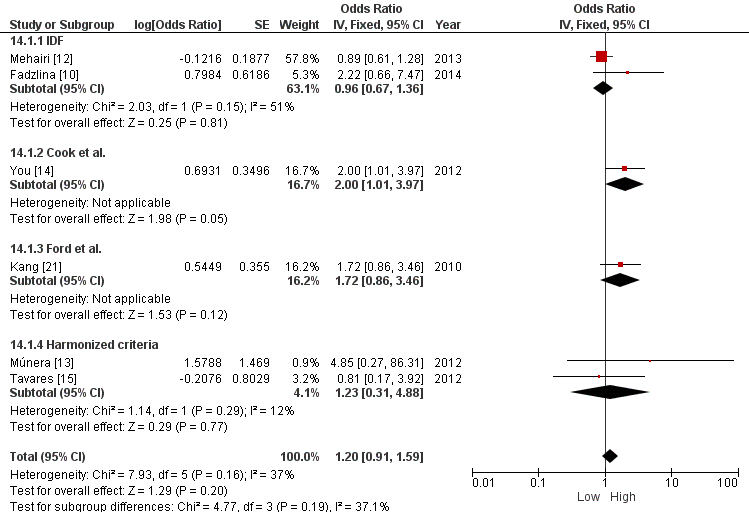

Supplement: S14 Fig — (PNG) [file pone.0168503.s014.png]

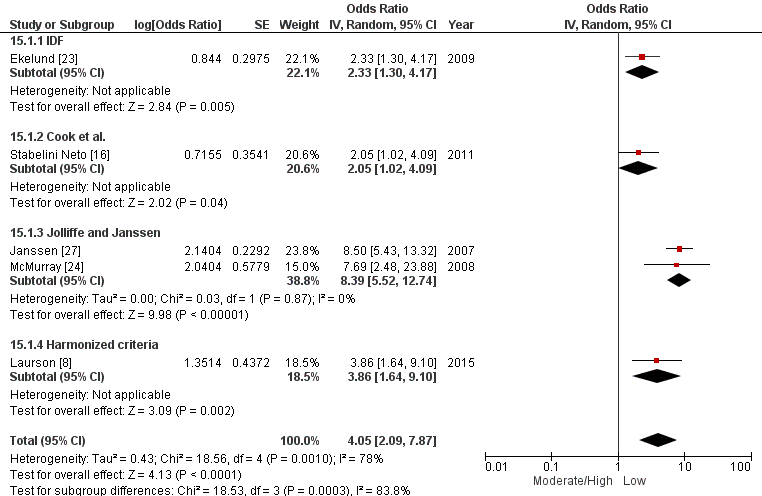

Supplement: S15 Fig — (PNG) [file pone.0168503.s015.png]

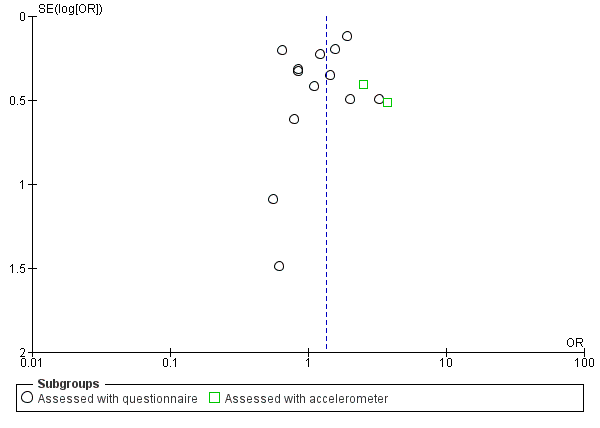

Supplement: S16 Fig — (PNG) [file pone.0168503.s016.png]
