# Supplementary material for: Physical Activity, Sedentary Behavior, Cardiorespiratory Fitness and Metabolic Syndrome in Adolescents: Systematic Review and Meta-Analysis of Observational Evidence
Source: PLoS One. 2016 Dec 20;11(12):e0168503. doi: 10.1371/journal.pone.0168503 (PMC5173371; doi:10.1371/journal.pone.0168503)
Supplement: S2 Table — (DOCX) [file pone.0168503.s018.docx]

**S2 Table. Studies excluded after reading the full text**

| **First author and year** | **Title** | **Journal, volume, issue and page** | **Reason for exclusion*** |
| --- | --- | --- | --- |
| Booth  (2005) | Methods of the NSW Schools Physical Activity and Nutrition Survey (SPANS) | J Sci Med Sport.  8(3):284-293 | 1 |
| Countryman  (2013) | Cardiometabolic risk in adolescents: associations with physical activity, fitness, and sleep--in response to Kawada | Ann Behav Med. 46(3):403-404 | 1 |
| Kawada  (2013) | Sleep duration, physical activity, and metabolic syndrome for adolescents | Ann Behav Med. 46(3):401-402 | 1 |
| Maia  (2013) | Physical activity, physical fitness, gross motor coordination, and metabolic syndrome: focus of twin research in Portugal | Twin Res Hum Genet. 16(1):296-301 | 1 |
| Shaibi (2008) | Cardiorespiratory fitness is strongly related to the metabolic syndrome in adolescents: response to Janssen and Cramp | Diabetes Care. 31(2):e8; author reply e9 | 1 |
| Lobelo (2007) | Cardiorespiratory fitness as criterion validity for health-based metabolic syndrome definition in adolescents | J Am Coll Cardiol. 31;50(5):471-2 | 1 |
| González  (2012) | Inflammatory markers and metabolic syndrome among adolescents | Eur J Clin Nutr. 66(10):1141-1145 | 2 |
| Haas  (2012) | Metabolic risk factors, leisure time physical activity, and nutrition in german children and adolescents | Cholesterol. 2012:370850 | 2 |
| Kelishadi  (2009) | Factors associated with insulin resistance and non-alcoholic fatty liver disease among youths | Atherosclerosis. 204(2):538-543 | 2 |
| Mazza  (2011) | Estudio clínico del síndrome metabólico en niños  y adolescentes de argentina | Rev Argent Salud Pública. 2(6):25-33 | 2 |
| Ventura  (2006) | Risk profiles for metabolic syndrome in a nonclinical sample of adolescent girls | Pediatrics.118(6):2434-2442 | 2 |
| Walker  (2012) | Racial/ethnic discrepancies in the metabolic syndrome begin in childhood and persist after adjustment for environmental factors | Nutr Metab Cardiovasc Dis. 22(2):141-148 | 2 |
| Wicklow  (2012) | Metabolic consequences of hepatic steatosis in overweight and obese adolescents | Diabetes Care. 35(4):905-910 | 2 |
| Vergetaki  (2011) | Presence of metabolic syndrome and cardiovascular risk factors in adolescents and University students in Crete (Greece), according to different levels of snack consumption | Appetite. 57(1):278-85 | 2 |
| Bitsori  (2009) | Waist circumference as a screening tool for the identification of adolescents with the metabolic syndrome phenotype | Int J Pediatr Obes. 4(4):325-31 | 2 |
| Bortoloti  (2015) | Prevalence of metabolic syndrome and associated factors in 11- to 17-year-old adolescents | Rev. Bras. Cineantropom. Desempenho Hum. 17(6):683-692 | 2 |
| Lee (2014) | Relation between sleep duration, overweight, and metabolic syndrome in Korean adolescents | Nutr Metab Cardiovasc Dis. 24(1):65-71 | 2 |
| Sung (2011) | Does sleep duration predict metabolic risk in obese adolescents attending tertiary services? A cross-sectional study | Sleep. 1;34(7):891-8 | 2 |
| Welk (2011) | Development of youth aerobic-capacity standards using receiver operating characteristic curves | Am J Prev Med. 41(4 Suppl 2):S111-6. | 2 |
| Flouris (2008) | Longitudinal preventive-screening cutoffs for metabolic syndrome in adolescents | Int J Obes (Lond). 32(10):1506-12. | 2 |
| Li  (2005) | Disease risks of childhood obesity in China | Biomed Environ Sci. 18(6):401-410 | 2 |
| Andersen  (2003) | Biological cardiovascular risk factors cluster in Danish children and adolescents: the European Youth Heart Study | Prev Med. 37(4):363-367 | 3 |
| Andersen  (2004) | The relationship between physical fitness and clustered risk, and tracking of clustered risk from adolescence to young adulthood: eight years follow-up in the Danish Youth and Sport Study | Int J Behav Nutr Phys Act. 8;1(1):6 | 3 |
| Countryman  (2013) | Cardiometabolic risk in adolescents: associations with physical activity, fitness, and sleep | Ann Behav Med. 45(1):121-131 | 3 |
| Eisenmann  (2004) | Stability of variables associated with the metabolic syndrome from adolescence to adulthood: the Aerobics Center Longitudinal Study | Am J Hum Biol. 16(6):690-696 | 3 |
| Ferreira  (2005) | Development of fatness, fitness, and lifestyle from adolescence to the age of 36 years: determinants of the metabolic syndrome in young adults: the amsterdam growth and health longitudinal study | Arch Intern Med. 10;165(1):42-48 | 3 |
| Hong  (2009) | Physical activity and metabolic syndrome in Korean children | Int J Sports Med. 30(9):677-683 | 3 |
| Kelishadi  (2013) | First report on path analysis for cardiometabolic components in a nationally representative sample of pediatric population in the Middle East and North Africa (MENA): the CASPIAN-III Study | Ann Nutr Metab. 62(3):257-265 | 3 |
| Kuk  (2010) | Independent associations between cardiorespiratory fitness and abdominal obesity with metabolic risk in adolescents and adults | Obesity (Silver Spring). 18(10):2061-2063 | 3 |
| Martínez-Gómez  (2009) | The role of physical activity and fitness on the metabolic syndrome in adolescents: effect of different scores. The AFINOS Study | J Physiol Biochem. 65(3):277-289 | 3 |
| Molero-Conejo  (2003) | Lean adolescents with increased risk for metabolic syndrome | Arch Latinoam Nutr. 53(1):39-46 | 3 |
| Moreira  (2011) | Metabolic risk factors, physical activity and physical fitness in Azorean adolescents: a cross-sectional study | BMC Public Health. 11:214 | 3 |
| Pinto  (2011) | Parâmetros metabólicos e fatores de risco associados à obesidade abdominal em adolescentes do sexo feminino de escolas públicas do Distrito Federal (Brasil) | Arch latinoam nutr. 61(1):55-64 | 3 |
| Santos  (2013) | Genotype by energy expenditure interaction with metabolic syndrome traits: the Portuguese healthy family study | PLoS One. 18;8(11):e80417 | 3 |
| Sénéchal  (2013) | Cardiorespiratory fitness and adiposity in metabolically healthy overweight and obese youth | Pediatrics. 132(1):e85-92 | 3 |
| Török  (2001) | Low physical performance in obese adolescent boys with metabolic syndrome | Int J Obes Relat Metab Disord. 25(7):966-970 | 3 |
| Twig  (2014) | Cardiovascular and metabolic risk factors in inherited autoinflammation | J Clin Endocrinol Metab. 99(10):E2123-8 | 3 |
| Ventura  (2009) | Developmental trajectories of girls' BMI across childhood and adolescence | Obesity (Silver Spring). 17(11):2067-2074 | 3 |
| Bacha  (2006) | Are obesity-related metabolic risk factors modulated by the degree of insulin resistance in adolescents? | Diabetes Care. 29(7):1599-604 | 3 |
| Wennberg (2013) | Television viewing and low leisure-time physical activity in adolescence independently predict the metabolic syndrome in mid-adulthood | Diabetes Care. 2013 Jul;36(7):2090-7 | 3 |
| Jekal  (2014) | The association of adolescent fatness and fitness with risk factors for adult metabolic syndrome: a 22-year follow-up study | J Phys Act Health. 11(4):823-830 | 3 |
| Buff  (2007) | Freqüência de síndrome metabólica em crianças e adolescentes com sobrepeso e obesidade | Rev Paul Pediatr. 25(3): 221-226 | 4 |
| Butte  (2007) | Physical activity in nonoverweight and overweight Hispanic children and adolescents | Med Sci Sports Exerc. 39(8):1257-1266 | 4 |
| Santos  (2013) | Physical activity, fitness and the metabolic syndrome in rural youths from Mozambique | Ann Hum Biol. 40(1):15-22 | 4 |
| Gardner  (2013) | Metabolic syndrome and daily ambulation in children, adolescents, and young adults | Med Sci Sports Exerc. 45(1):163-169 | 4 |
| Hsu  (2011) | Physical activity, sedentary behavior, and the metabolic syndrome in minority youth | Med Sci Sports Exerc. 43(12):2307-2313 | 4 |
| Hsu  (2014) | Double jeopardy: metabolic syndrome leads to increased sedentary behavior in peri-pubertal minority females | Pediatr Exerc Sci. 26(3):266-273 | 4 |
| Janssen  (2013) | The fractionalization of physical activity throughout the week is associated with the cardiometabolic health of children and youth | BMC Public Health. 6;13:554 | 4 |
| Jääskeläinen  (2012) | Childhood nutrition in predicting metabolic syndrome in adults: the cardiovascular risk in Young Finns Study | Diabetes Care. 35(9):1937-1943 | 4 |
| Kelishadi  (2007) | Association of physical activity and the metabolic syndrome in children and adolescents: CASPIAN Study | Horm Res. 67(1):46-52 | 4 |
| Kelishadi  (2008) | Factors associated with the metabolic syndrome in a national sample of youths: CASPIAN Study | Nutr Metab Cardiovasc Dis. 18(7):461-470 | 4 |
| Maligie  (2012) | Adiposity, fat patterning, and the metabolic syndrome among diverse youth: the EPOCH study | J Pediatr. 161(5):875-880 | 4 |
| McMurray  (2011) | Importance of proper scaling of aerobic power when relating to cardiometabolic risk factors in children | Ann Hum Biol. 38(5):647-654 | 4 |
| Moore  (2008) | Physical activity, metabolic syndrome, and overweight in rural youth | J Rural Health. 24(2):136-142 | 4 |
| Monzani  (2014) | Metabolic syndrome is strictly associated with parental obesity beginning from childhood | Clin Endocrinol (Oxf). 81(1):45-51 | 4 |
| Pedrozo  (2008) | Síndrome metabólico y factores de riesgo asociados con el estilo de vida de adolescentes de una ciudad de Argentina, 2005 | Rev Panam Salud Publica. 24(3):149-160 | 4 |
| Sisson  (2013) | Sitting and cardiometabolic risk factors in U.S. adolescents | J Allied Health. 42(4):236-242 | 4 |
| Tangeraas  (2010) | Cardiorespiratory fitness is a marker of cardiovascular health in renal transplanted children | Pediatr Nephrol. 25(11):2343-2350 | 4 |
| Yang  (2009) | Sustained participation in youth sport decreases metabolic syndrome in adulthood | Int J Obes (Lond). 33(11):1219-1226 | 4 |
| Enes Romero  (2013) | Influencia étnica en la prevalencia de síndrome metabólico en población pediátrica obesa | An Pediatr (Barc). 78(2):75-80 | 4 |
| Leskinen  (2009) | Effects of 32-year leisure time physical activity discordance in twin pairs on health (TWINACTIVE study): aims, design and results for physical fitness | Twin Res Hum Genet. 12(1):108-17 | 4 |
| Conus  (2004) | Metabolic and behavioral characteristics of metabolically obese but normal-weight women | J Clin Endocrinol Metab. 89(10):5013-20 | 4 |
| Ma  (2010) | Associations of Fibrinogen with Metabolic Syndrome in Rural Chinese Population | J Ather Thromb. 17(5):486-492 | 4 |
| Andersen (2015) | A new approach to define and diagnose cardiometabolic disorder in children | J Diabetes Res. 2015:539835 | 4 |
| Briggs (2015) | Cardiovascular risk and metabolic syndrome in obese youth enrolled in a multidisciplinary medical weight management program: implications of musculoskeletal pain, cardiorespiratory fitness, and health-related quality of life | Metab Syndr Relat Disord. 13(3):102-9 | 4 |
| Sutherland (2014) | The relationship of metabolic syndrome and health-promoting lifestyle profiles of Latinos in the Northwest | Hisp Health Care Int. 12(3):130-7 | 4 |

*Reason for exclusion: 1- Study design; 2- Metabolic Syndrome not linked with outcomes of interest; 3- Did not consider Metabolic Syndrome; 4- Age
