# Supplementary material for: Physical Activity, Sedentary Behavior, Cardiorespiratory Fitness and Metabolic Syndrome in Adolescents: Systematic Review and Meta-Analysis of Observational Evidence
Source: PLoS One. 2016 Dec 20;11(12):e0168503. doi: 10.1371/journal.pone.0168503 (PMC5173371; doi:10.1371/journal.pone.0168503)
Supplement: S3 Table — (DOCX) [file pone.0168503.s019.docx]

**S3 Table. Methodological quality of the 21 studies included in the systematic review***

| **Study** | **Is the hypothesis/ aim/ objective of the study clearly described?** | **Are the main outcomes to be measured clearly described in the Introduction or Methods section?** | **Are the characteristics of the patients included in the study clearly described?** | **Are the main findings of the study clearly described?** | **Does the study provide estimates of the random variability in the data for the main outcomes?** | **Have actual probability values been reported (e.g.0.035 rather than <0.05) for the main outcomes except where the probability value is less than 0.001?** | **Were those subjects who were prepared to participate representative of the entire population from which they were recruited?** | **If any of the results of the study were based on “data dredging”, was this made clear?** | **Were the main outcome measures used accurate (valid and reliable)?** | **Was there adequate adjustment for confounding in the analyses from which the main findings were drawn?** | **Score** |
| --- | --- | --- | --- | --- | --- | --- | --- | --- | --- | --- | --- |
| Bermúdez-Cardona [7] (2016) | Yes | Yes | No | Yes | Yes | Yes | No | Yes | Yes | Yes | 8 |
| Laurson [8] (2015) | Yes | Yes | No | No | Yes | No | Yes | Yes | Yes | No | 6 |
| Rafraf [9] (2014) | No | Yes | Yes | Yes | Yes | Yes | No | Yes | Yes | Yes | 8 |
| Fadzlina [10] (2014) | Yes | No | Yes | No | No | No | Yes | No | No | Yes | 4 |
| Fam [11] (2013) | Yes | Yes | Yes | Yes | Yes | Yes | No | Yes | Yes | Yes | 9 |
| Mehairi [12] (2013) | Yes | No | Yes | No | Yes | Yes | No | Yes | Yes | No | 6 |
| Múnera [13] (2012) | Yes | Yes | Yes | Yes | Yes | Yes | No | Yes | Yes | Yes | 9 |
| You [14] (2012) | Yes | Yes | Yes | Yes | Yes | Yes | Yes | Yes | No | Yes | 9 |
| Tavares [15] (2012) | Yes | Yes | Yes | Yes | Yes | Yes | No | Yes | Yes | Yes | 9 |
| Stabelini Neto [16] (2011) | Yes | Yes | Yes | Yes | Yes | No | Yes | Yes | Yes | Yes | 9 |
| Mikołajczak [17] (2011) | Yes | No | No | Yes | Yes | No | No | Yes | No | Yes | 5 |
| Aboul Ella [18] (2010) | Yes | Yes | Yes | Yes | Yes | Yes | No | No | No | Yes | 7 |
| Nguyen [19] (2010) | Yes | Yes | Yes | Yes | Yes | Yes | No | Yes | Yes | Yes | 9 |
| Budak [20] (2010) | Yes | Yes | Yes | Yes | Yes | Yes | Yes | Yes | No | No | 8 |
| Kang [21] (2010) | Yes | Yes | Yes | Yes | Yes | Yes | Yes | Yes | Yes | Yes | 10 |
| Moreira [22] (2010) | Yes | Yes | Yes | Yes | Yes | Yes | No | Yes | Yes | Yes | 9 |
| Ekelund [23] (2009) | Yes | Yes | Yes | Yes | Yes | Yes | No | Yes | Yes | Yes | 9 |
| McMurray [24] (2008) | Yes | Yes | Yes | Yes | Yes | Yes | No | Yes | Yes | Yes | 9 |
| Mark [25] (2008) | Yes | Yes | Yes | Yes | Yes | Yes | No | Yes | No | Yes | 8 |
| Pan [26] (2008) | Yes | Yes | Yes | Yes | Yes | No | Yes | No | No | Yes | 7 |
| Janssen [27] (2007) | Yes | Yes | No | No | Yes | No | Yes | Yes | Yes | Yes | 7 |

*Adapted version of the tool proposed by Downs and Black [36]
