# Supplementary material for: Physical Activity, Sedentary Behavior, Cardiorespiratory Fitness and Metabolic Syndrome in Adolescents: Systematic Review and Meta-Analysis of Observational Evidence
Source: PLoS One. 2016 Dec 20;11(12):e0168503. doi: 10.1371/journal.pone.0168503 (PMC5173371; doi:10.1371/journal.pone.0168503)
Supplement: S4 Table — (DOCX) [file pone.0168503.s020.docx]

**S4 Table. Metabolic syndrome events in different classifications of physical activity**

| **Study** | **Low** | | **Moderate/High** | |
| --- | --- | --- | --- | --- |
|  | **Total**  **N** | **Events**  **N (%)** | **Total**  **N** | **Events**  **N (%)** |
| Bermúdez-Cardona [7] | - | - | - | - |
| Rafraf [9] | 6 | 0 (0.0) | 210 | 23 (10.9) |
| Fadzlina [10] | 642 | 17 (2.6) | 372 | 9 (2.4) |
| Fam [11] | - | - | - | - |
| Mehairi [12] | 350 | 36 (10.3) | 648 | 97 (15.0) |
| Múnera [13] | 51 | 1 (2.0) | 174 | 6 (3.4) |
| You [14] | 342 | 37 (10.8) | 264 | 43 (16.3) |
| Tavares [15] | 159 | 10 (6.3) | 51 | 4 (7.8) |
| Stabelini Neto [16] | 183 | 17 (9.3) | 273 | 18 (6.6) |
| Mikołajczak [17] | 299 | 19 (6.4) | 479 | 36 (7.5) |
| Aboul Ella [18] | - | - | - | - |
| Nguyen [19] | 249 | 18 (7.2) | 246 | 5 (2.0) |
| Budak [20] | - | - | - | - |
| Ekelund [23] | - | - | - | - |
| McMurray [24] | 131 | 11 (8.4) | 258 | 7 (2.7) |
| Mark [25] | - | - | - | - |
| Pan [26] | 1037 | 45 (4.3) | 2420 | 68 (2.8) |

- no data available.
