# Supplementary material for: Physical Activity, Sedentary Behavior, Cardiorespiratory Fitness and Metabolic Syndrome in Adolescents: Systematic Review and Meta-Analysis of Observational Evidence
Source: PLoS One. 2016 Dec 20;11(12):e0168503. doi: 10.1371/journal.pone.0168503 (PMC5173371; doi:10.1371/journal.pone.0168503)
Supplement: S5 Table — (DOCX) [file pone.0168503.s021.docx]

**S5 Table. Metabolic syndrome events in different classifications of sedentary behavior**

| **Study** | **High** | | **Low** | |
| --- | --- | --- | --- | --- |
|  | **Total**  **N** | **Events**  **N (%)** | **Total**  **N** | **Events**  **N (%)** |
| Bermúdez-Cardona [7] | - | - | - | - |
| Fadzlina [10] | 789 | 23 | 225 | 3 |
| Mehairi [12] | 534 | 67 | 459 | 64 |
| Múnera [13] | 172 | 7 | 53 | 0 (0.0) |
| You [14] | 358 | 57 | 248 | 23 |
| Tavares [15] | 170 | 10 | 28 | 2 |
| Kang [21] | 640 | 52 | 205 | 10 |
| Ekelund [23] | - | - | - | - |
| Mark [25] | - | - | - | - |

- no data available.
