# Supplementary material for: Physical Activity, Sedentary Behavior, Cardiorespiratory Fitness and Metabolic Syndrome in Adolescents: Systematic Review and Meta-Analysis of Observational Evidence
Source: PLoS One. 2016 Dec 20;11(12):e0168503. doi: 10.1371/journal.pone.0168503 (PMC5173371; doi:10.1371/journal.pone.0168503)
Supplement: S6 Table — (DOCX) [file pone.0168503.s022.docx]

**S6 Table. Metabolic syndrome events in different classifications of cardiorespiratory fitness**

| **Study** | **Low** | | **Moderate/High** | |
| --- | --- | --- | --- | --- |
|  | **Total**  **N** | **Events**  **N (%)** | **Total**  **N** | **Events**  **N (%)** |
| Laurson [8] | 122 | 15 (12.3) | 257 | 9 (3.5) |
| Stabelini Neto [16] | 150 | 17 (11.3) | 306 | 18 (5.9) |
| Moreira [22] | 305 | 26 (8.5) | 212 | 0 (0.0) |
| Ekelund [23] | - | - | - | - |
| McMurray [24] | 130 | 14 (10.8) | 259 | 4 (1.5) |
| Janssen [27] | 520 | 93 (17.9) | 1041 | 26 (2.5) |

- no data available.
